# Granular cell tumor of the oral cavity; a case series including a case of metachronous occurrence in the tongue and the lung

**DOI:** 10.4317/medoral.19867

**Published:** 2014-06-01

**Authors:** Sander van de Loo, Erik Thunnissen, Pieter Postmus, Isaäc van der Waal

**Affiliations:** 1 Dept. of Oral and Maxillofacial Surgery/Pathology, VU university medical center/ Academic Centre for Dentistry Amsterdam (ACTA), Amsterdam; 2 Dept. of Pathology, VU university medical center, Amsterdam; 3 Dept. of Pulmonology, VU university medical center, Amsterdam, The Netherlands

## Abstract

The granular cell tumor (GCT) is a rare, benign tumor that most commonly occurs in the oral cavity, particularly in the anterior part of the tongue. In this study the experience with 16 patients with a GCT observed in a single Institution will be discussed. Although no radicality has been obtained in most cases, recurrences are rare. In one patient, a recurrence was noted four years after excision of the primary. In the same patient a pulmonary lesion occurred five years after excision of the recurrence in the oral cavity, most likely representing an example of metachronous occurrence and not a distant metastasis. Since recurrences and metachronous lesions are rare, as are distant metastases, routine follow-up does not seem warranted in patients treated for a granular cell tumor of the oral cavity.

** Key words:**Granular cell tumor, mouth, tongue, metachronous occurrence, metastasis.

## Introduction

Granular cell tumor (GCT) is a soft tissue neoplasm first reported in 1926 by Abrikossoff and for a long time considered to be of myoblastic origin (“granular cell myoblastoma”) (1). At present, the lesion is believed to arise from Schwann cells. GCT does rarely metastasize; some 100 of such cases have been reported, the majority being primarily localized on the lower extremities. (2) 

Granular cell tumors may occur everywhere in the body but do have a preference for the tongue. Apparently, there is a slight female predilection. The lesion most frequently occurs in the fourth to sixth decades and is rare in children. The usual clinical presentation is an otherwise asymptomatic solitary nodule on the anterior part of the tongue, sometimes having a yellowish or pinkish appearance; multiple, simultaneously occurring GCTs are rare.

Histopathologically, the lesion is rather well-circumscribed but not truly encapsulated. The lesional cells are polygonal and show numerous cytoplasmatic granules. The cytoplasm stains positive immunohistochemically for S-100 protein. There is absence of nuclear and cellular pleomorphism and mitotic activity is rarely observed. A well known phenomenon in GCT is the presence of pseudoepitheliomatous hyperplasia (PEH) of the overlying epithelium. Such PEH may be misdiagnosed as a squamous cell carcinoma in case of a small biopsy.

Treatment consists of conservative excision. Although radicality as assessed by histopathological examination may not be obtained in all cases, recurrences are rare.

The purpose of this treatise is to present a single institution experience with 16 cases of oral GCT.

## Patient and Methods

In the period between 1977 and 2013 20 cases of previously untreated GCT of the oral cavity could be retrieved from the files of the department of Oral and Maxillofacial Surgery/Pathology of the VU university medical center in Amsterdam, the Netherlands. In four patients no histopathological slides were available for review. These cases have been deleted from the study. The data of the remaining 16 patients are presented in  Table 1. In all patients an excisional biopsy has been performed. No structured follow-up schedule had been arranged in these patients.

**Table 1:**
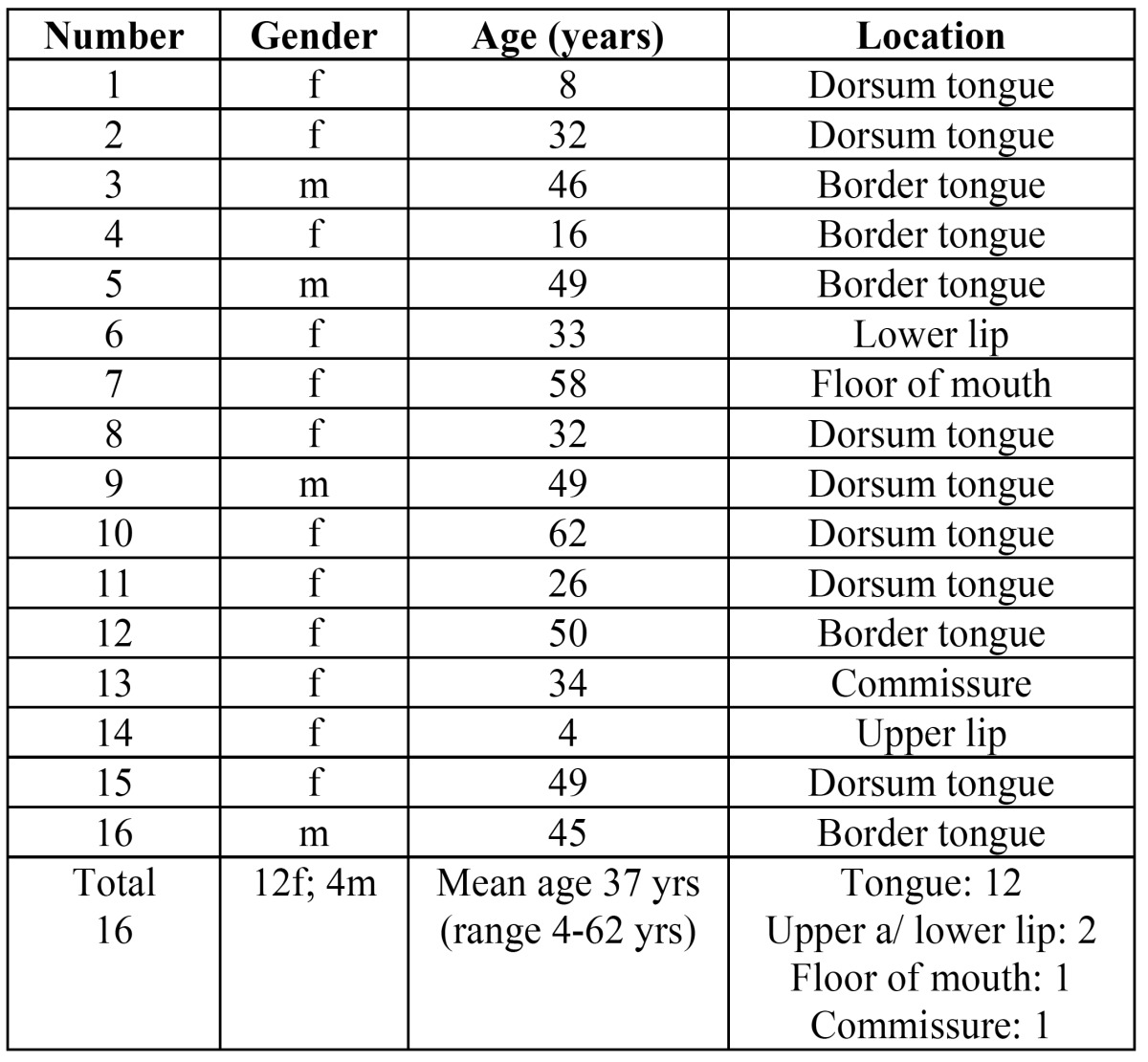
Demographic and clinical data of 16 patients with an oral granular cell tumor.

The design of this retrospective study adheres to the code for proper use of human material of the Dutch Federation of Biomedical Societies (http://www.federa.org).

## Results

**Figure 1:**
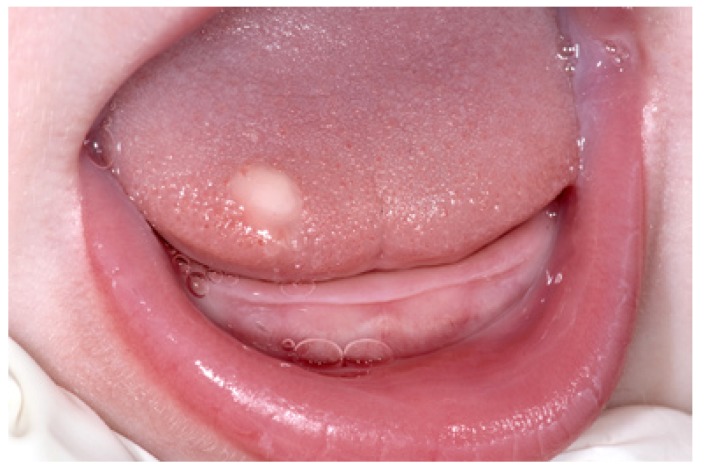
Granular cell tumor of the tongue in a 5-months-old baby.

As is shown in table 1 there was a threefold preponderance of females in this series. The mean age amounted 37 years (range 4 to 62 years). Remarkably, two patients were diagnosed in their first decade. In 12 patients the GCT was located on the tongue, most commonly on the dorsum of the anterior part of the tongue (Fig. 1). One lesion was located in the floor of the mouth and the remaining three lesions were located on the upper lip, the lower lip (Fig. 1), and the commissure, respectively. All patients presented with a solitary lesion.

**Figure 2:**
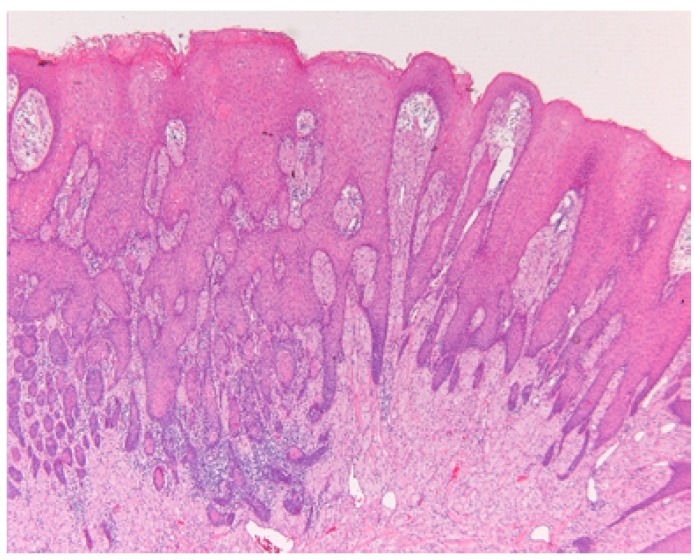
Pseudoepitheliomatous hyperplasia overlying a granular cell tumor of the tongue.

**Figure 3:**
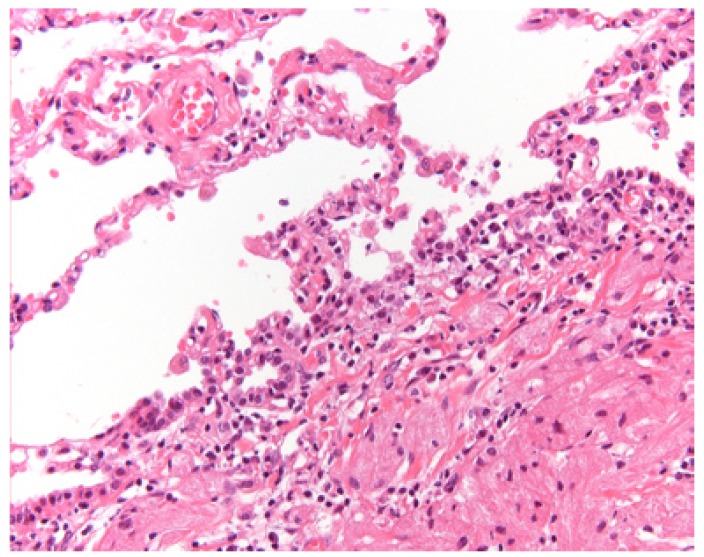
CT showing involvement of the right lower lobe in patient # 8.

In all patients the lesion has been excised. Pseudoepitheliomatous hyperplasia of the overlying epithelium was a common finding (Fig. 2). A few cases were stained immunohistochemically with S-100 protein, being positive in all such cases. Histopathologically, radicality was rarely obtained. Nevertheless, a recurrence was observed in only one patient (patient #8) four years after the first removal. Five years after removal of the recurrent lesion of the dorsal surface of the tongue the patient developed a GCT in the right lower lobe of the lung around the bronchus (Fig. 3). The patient underwent a lobectomy. Histologically, no signs of malignancy were observed in the pulmonary tumor (Fig. 3). There was low proliferative activity as observed in the MIB1 immunohistochemical stain. In the surrounding lymph nodes no tumor cells were seen. Comparative genomic hybridization (CGH) analysis did not show distinct gains or losses in either the tongue lesion or the pulmonary lesion.

## Discussion

The rarity of GCT of the oral cavity is well demonstrated by the low number of 16 cases during a period of some 35 years in a single university department of oral and maxillofacial surgery. The estimated incidence of oral CGT is approximately 1: 1.000,000 population per year. There are no distinct geographical or racial differences. Apparently, there is no explanation for the well known female predilection. GCT may occur already at an early age; even congenital occurrence has been reported (2). The clinical presentation of a small, firm yellowish nodule on the tongue is rather characteristic and almost pathognomonic of a GCT. Nevertheless, a biopsy, usually an excisional one, is indicated. In most instances no radicality has been obtained at the microscopic level after surgical excision, raising the issue of the need for a re-excision and the extent of such re-excision. In none of our patients a re-excision has been performed. In one patient the lesion recurred four years after removal of the primary lesion. In view of the rather limited number of patients in our series and also in view of the lack of a structured follow-up schedule we feel not justified to make any strong recommendations in this respect.

Synchronous multiple oral GCTs are rare (3). Whether metachronous GCTs are to be regarded a second primary or a distant metastasis could not be assessed in the present case on histopathological criteria. In the absence of lack of gains and losses in the CGH analysis possible clonality could not be demonstrated in our patient with pulmonary involvement. Based on the specific location: submucosal in the bronchial wall and in view of the absence of lymph node metastases, lack of mitoses and cytonuclear atypia, low MIB1 positivity (<5%), we have most likely been dealing with a metachronous primary pulmonary lesion rather than a metastatic one. The three year follow-up in this patient after the lobectomy has been uneventful. Remarkably, the patient has been treated for a melanoma of one of the toes two years before she experienced the first GCT in her tongue. To the best of our knowledge, no association between the occurrence of melanomas and GCTs is known from the literature.

Apparently 1%-2% of histologically benign GCTs may metastasize, commonly through the hematogenous route. Several criteria of malignancy of CGT have been suggested, such as those proposed by Fanburg-Smith ( Table 2) (1). The common sites for metastases are the bones, regional lymph nodes, peripheral nerves, the peritoneal cavity, the breast and the lung (4). In the reported cases several years evolved between initial treatment and the occurrence of metastases. At the same, it is well known that benign GCTs may also arise primarily in the lungs (5,6). The histologic criteria of malignancy proposed by Nasser H. are still debatable amongst pathologists (7).

**Table 2:**
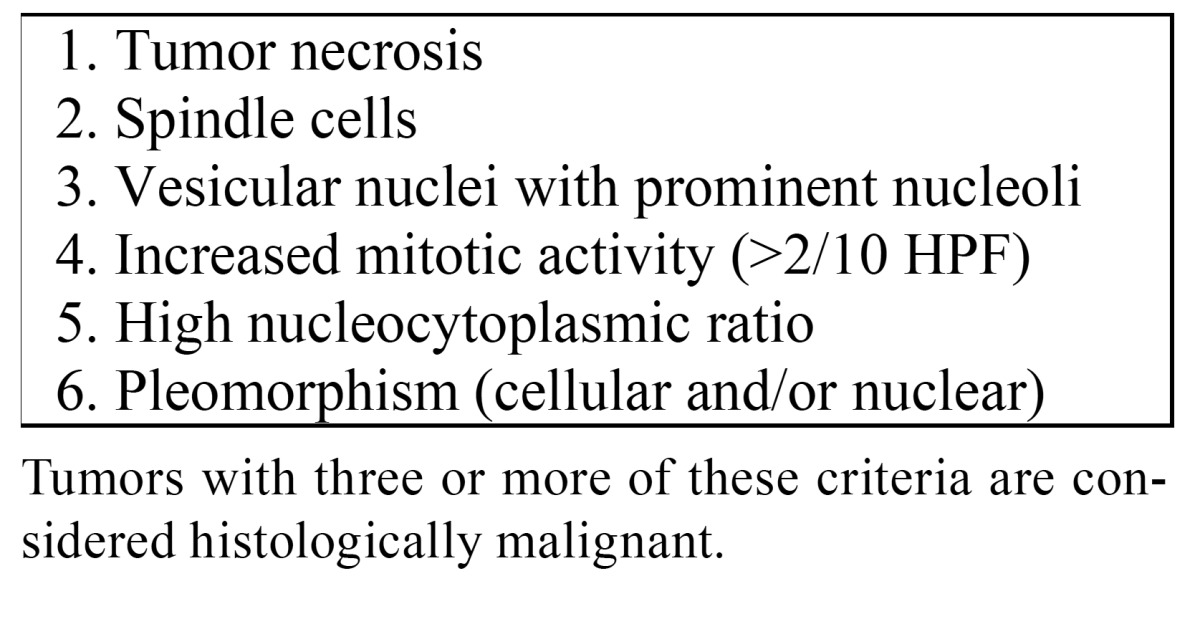
Criteria of histologic malignancy of GCT proposed by Fanburg-Smith (1).

Brandwein. recognize three types of malignant granular cell tumors: 1) histologically and biologically malignant, 2) histologically benign but biologically malignant, and 3) histologically atypical, clinically aggressive yet not metastatic (8).

According to Jardines. multi focal CGTs have to be considered as malignant (9). Nevertheless, there remains room for discussion whether or multifocality is based on metastatic spread, indeed (10).

Apparently, there is no specific karyotype that characterizes the GCT. Although, loss of heterozygosity on chromosomes 9p and 17p has been reported, this was not detectable in the CGH analysis (11).

Pseudoepitheliomatous hyperplasia in the overlying stratified squamous epithelium of a GCT is a well known, yet poorly understood phenomenon. A few cases have been reported of the simultaneous presence of a squamous cell carcinoma of the tongue and a GCT showing pseudoepitheliomatous hyperplasia (12,13).

Surgery is the treatment of choice. Adjuvant radiotherapy may be considered for recurrent lesions.; Apparently, there is no role for chemotherapy (14).

Overall, routine follow-up does not seem warranted in patients treated for a granular cell tumor of the oral ca-vity.
